# Food waste within food supply chains: quantification and potential for change to 2050

**DOI:** 10.1098/rstb.2010.0126

**Published:** 2010-09-27

**Authors:** Julian Parfitt, Mark Barthel, Sarah Macnaughton

**Affiliations:** 1 Resource Futures, Bristol, UK; 2 Waste and Resources Action Programme, Banbury, UK; 3 Isis Innovation Ltd, Oxford, UK

**Keywords:** food waste, post-harvest loss, consumer waste

## Abstract

Food waste in the global food supply chain is reviewed in relation to the prospects for feeding a population of nine billion by 2050. Different definitions of food waste with respect to the complexities of food supply chains (FSCs)are discussed. An international literature review found a dearth of data on food waste and estimates varied widely; those for post-harvest losses of grain in developing countries might be overestimated. As much of the post-harvest loss data for developing countries was collected over 30 years ago, current global losses cannot be quantified. A significant gap exists in the understanding of the food waste implications of the rapid development of ‘BRIC’ economies. The limited data suggest that losses are much higher at the immediate post-harvest stages in developing countries and higher for perishable foods across industrialized and developing economies alike. For affluent economies, post-consumer food waste accounts for the greatest overall losses. To supplement the fragmentary picture and to gain a forward view, interviews were conducted with international FSC experts. The analyses highlighted the scale of the problem, the scope for improved system efficiencies and the challenges of affecting behavioural change to reduce post-consumer waste in affluent populations.

## 1. Introduction

Attempts have been made to quantify global food waste over several decades, motivated partly by the need to highlight the scale of ‘waste’ in relation to global malnutrition. Such assessments are reliant on limited datasets collected across the food supply chain (FSC) at different times and extrapolated to the larger picture. The most often quoted estimate is that ‘as much as half of all food grown is lost or wasted before and after it reaches the consumer’ (Lundqvist *et al*. 2008). Such estimates are difficult to scrutinize but highlight the need for greater resource efficiencies in the global FSC. This paper presents results from a driver review of food waste issues, combining information on food waste from the international literature and interviews with supply chain experts.

### (a) Definitions

Although waste is formally defined in different legal jurisdictions, definitions relate to particular points of arising and are often framed in relation to specific environmental controls. Food waste occurs at different points in the FSC, although it is most readily defined at the retail and consumer stages, where outputs of the agricultural system are self-evidently ‘food’ for human consumption. Unlike most other commodity flows, food is biological material subject to degradation, and different food stuffs have different nutritional values. There are also moral and economic dimensions: the extent to which available food crops are used to meet global human needs directly, or diverted into feeding livestock, other ‘by-products’ and biofuels or biomaterials production. Below are three definitions referred to herein:

1. Wholesome edible material intended for human consumption, arising at any point in the FSC that is instead discarded, lost, degraded or consumed by pests (FAO 1981).
2. As (1), but including edible material that is intentionally fed to animals or is a by-product of food processing diverted away from the human food (Stuart 2009).
3. As definitions (1) and (2) but including over-nutrition—the gap between the energy value of *consumed* food *per capita* and the energy value of food *needed per capita* (Smil 2004*a*).

The first two definitions are considered to be most relevant, although the second can only be supported if data are available that include nutritional assessment of animal feed and food processing by-products. Table 1 summarizes generalized stages in FSCs and illustrates different forms that ‘food waste’ may take.

**Table 1.** Generic FSC and examples of food waste.

	stage	examples of food waste/loss characteristics
(1)	harvesting—handling at harvest	edible crops left in field, ploughed into soil, eaten by birds, rodents, timing of harvest not optimal: loss in food quality
		crop damaged during harvesting/poor harvesting technique
		out-grades at farm to improve quality of produce
(2)	threshing	loss through poor technique
(3)	drying—transport and distribution	poor transport infrastructure, loss owing to spoiling/bruising
(4)	storage	pests, disease, spillage, contamination, natural drying out of food
	processing	
(5)	primary processing—cleaning, classification, de-hulling, pounding, grinding, packaging, soaking, winnowing, drying, sieving, milling	process losses
		contamination in process causing loss of quality
(6)	secondary processing—mixing, cooking, frying moulding, cutting, extrusion	process losses
		contamination in process causing loss of quality
(7)	product evaluation—quality control: standard recipes	product discarded/out-grades in supply chain
(8)	packaging—weighing, labelling, sealing	inappropriate packaging damages produce
		grain spillage from sacks
		attack by rodents
(9)	marketing—publicity, selling, distribution	damage during transport: spoilage
		poor handling in wet market
		losses caused by lack of cooling/cold storage
(10)	post-consumer—recipes elaboration: traditional dishes, new dishes product evaluation, consumer education, discards	plate scrapings
		poor storage/stock management in homes: discarded before serving
		poor food preparation technique: edible food discarded with inedible
		food discarded in packaging: confusion over ‘best before’ and ‘use by’ dates
(11)	end of life—disposal of food waste/loss at different stages of supply chain	food waste discarded may be separately treated, fed to livestock/poultry, mixed with other wastes and landfilled

Within the literature, food waste post-harvest is likely to be referred to as ‘food losses’ and ‘spoilage’. Food loss refers to the decrease in food quantity or quality, which makes it unfit for human consumption (Grolleaud 2002). At later stages of the FSC, the term food waste is applied and generally relates to behavioural issues. Food losses/spoilage, conversely, relate to systems that require investment in infrastructure. In this report, we refer to both food losses and food waste as food waste.

Similarly, both ‘FSC’ and ‘post-harvest systems’ are used to mean the same thing in the literature, with ‘post-harvest loss’ also often used when describing agricultural systems and the onward supply of produce to markets. FSC is more associated with industrialized countries where post-harvest processing and large retail sectors are important features. ‘Post-consumer losses’ include food wasted from activities and operations at the point at which food is consumed. The method of measuring the quantity of food post-harvest is usually by weight, although other units of measure include calorific value, quantification of greenhouse gas impacts and lost inputs (e.g. nutrients and water). Where loss data are available for each step of a crop and are applied to production estimates, a cumulative weight loss can be calculated.

## 2. Food waste in the supply chain

### (a) Introduction

When the Food and Agriculture Organization of the United Nations (FAO) was established in 1945, it had reduction of food losses within its mandate. By 1974, the first World Food Conference identified reduction of post-harvest losses as part of the solution in addressing world hunger. At this time, an overall estimate for post-harvest losses of 15 per cent had been suggested, and it was resolved to bring about a 50 per cent reduction by 1985. Consequently, the FAO established the Special Action Programme for the Prevention of Food Losses.

The main focus was initially on reducing losses of durable grain; by the early 1990s, the scope of work had been broadened to cover roots and tubers, and fresh fruits and vegetables (FFVs). Poor adoption rates for interventions led to the recognition that a purely technical focus was inadequate for solving problems within the sector and a more holistic approach was developed (Grolleaud 2002). There is no account of progress towards the 1985 post-harvest loss reduction target, and recently Lundqvist *et al*. (2008) called for action to reduce food waste advocating a 50 per cent reduction in post-harvest losses to be achieved by 2025.

### (b) Global trends that influence supply chain losses

Post-harvest losses are partly a function of the technology available in a country, as well as the extent to which markets have developed for agricultural produce. Three inter-related global drivers provide an overall structure for characterizing supply chains and future trends in developing and transitional countries.

— *Urbanization and the contraction of the agricultural sector*. The proportion of the world's population employed in agriculture has declined in recent decades and 50 per cent of the world's population now lives in urban environments. This proportion is expected to rise to 70 per cent by 2050 (United Nations 2008). Rapid urbanization has created the need for extended FSCs to feed urban populations. For these to be efficient, countries need improvements in roads, transportation and marketing infrastructure to keep food affordable for lower income groups. How these extended supply chains develop has implications for food waste globally, now and in the future.
— *Dietary transition*. Growth of household incomes, particularly in BRIC countries, is associated with a decline in consumption of starchy food staples and diversification of diet into FFVs, dairy, meat and fish. This transition conforms to Bennett's Law (Bennett 1941), where the food share of starchy staples declines as income increases. The shift towards vulnerable, shorter shelf-life items is associated with greater food waste and a greater draw on land and other resources (Lundqvist *et al*. 2008). The transition varies by country and culture, e.g. in India, there is less pressure on resources compared with China, where the demand for meat is increasing rapidly.
— *Increased globalization of trade*. International trade in processed foods accounts for 10 per cent of total processed food sold (United Nations 2002). Globalization may open up opportunities for agricultural exports while representing a threat to development of internal markets through competition from inexpensive imports of higher quality than can be produced locally. Linked to trade liberalization, multi-national chains have become a driving force in the rapid growth of supermarkets in many transitional economies.

Industrialized countries are experiencing other drivers, the most significant being the ageing population profile and growth in single person households.

**Figure 1.**
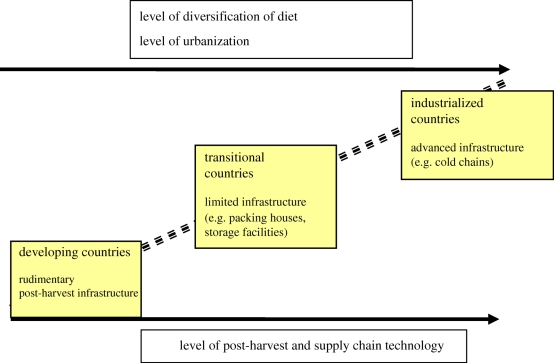
Schematic development of FSCs in relation to post-harvest infrastructure.

To reflect these important global drivers, post-harvest losses are considered along a technological/economic gradient: ‘developing’, ‘intermediate’ and ‘industrialized’ FSCs. Figure 1 provides an overview of the development of post-harvest infrastructure along this gradient, expanded in table 2.

**Table 2.** Characterization of post-harvest infrastructure in relation to stages of economic development.

type of post-harvest infrastructure	technological development	level of development	supply chain characteristics	type of growers	markets and quality
developing traditional systems	simple technologies, labour-intensive, traditional storage systems and harvesting techniques	low-income countries	poor integration with local markets, many intermediaries supplying urban markets	smallholders, including subsistence farmers	local markets: mostly meeting household/village food requirements; limited access to international markets
intermediate systems—‘transitional’	packing houses, refrigeration and storage facilities systems alongside elements of traditional systems	low- and middle-income countries	requires closer integration of growers, suppliers, processors and distribution systems	small-scale farmers who often have access to limited post-harvest-specific infrastructure	produce of variable quality, target both local (including supermarkets) and, increasingly, export markets in a number of countries
developed industrialized systems	access to relatively sophisticated technologies, e.g. packing-house equipment and cold chains; losses still occur; harvesting highly mechanized, e.g. wheat	middle- and high-income countries	use of highly integrated systems between growers and supply chain; more seasonal produce imported; more secondary processing of food	medium- and large-scale farmers	meet the quality and safety, as well as volume and timeliness demands of local (particularly supermarkets/convenience store chains) and export markets

*Developing countries*: The majority of the rural poor rely on short FSCs with limited post-harvest infrastructure and technologies. More extended FSCs feeding urban populations are likely to involve many intermediaries between growers and consumers, which may limit the potential for growers to receive higher prices for quality. Farming is mostly small scale with varying degrees of involvement in local markets and a rapidly diminishing proportion of subsistence farmers who neither buy nor sell food staples (Jayne *et al*. 2006). Interventions within these systems focus on training and upgrading technical capacity to reduce losses, increase efficiency and reduce labour intensity of the technologies employed. However, attempts to reduce post-harvest losses must take account of cultural implications. In years with food surpluses, the prices received for goods will be low. One option is to store surplus for lean years, but there may not be suitable storage facilities. To rectify this, investment and engineering skills are needed. There are many instances of relatively simple technologies providing effective solutions, such as an FAO project in Afghanistan and elsewhere that provided simple effective sealed storage drums for grain farmers, dramatically reducing post-harvest food losses (FAO 2008).

*Transitional and industrialized post-harvest systems* have a closer integration of producers, suppliers, processors, distribution systems and markets ensuring greater economies of scale, competitiveness and efficiency in the FSC. Supermarkets are the dominant intermediary between farmers and consumers. Even in poorer transitional economies, supermarkets are the main vehicle for delivering diversified diets: for the growing middle classes and the urban poor. This is almost entirely dependent on foreign direct investment, with high growth rates in Eastern Europe, Asia and Latin America (Reardon *et al*. 2007).

The sequence of transformation follows a different route in each country, particularly in the extent to which retailers bypass existing markets and traditional wholesalers to secure produce of the required standard and volume. There are often strong cross-links with export quality assurance, the quality standards set by supermarkets, and the procurement systems. Many of the issues identified are no different from supply chain issues in developed economies:

— payment terms discouraging small growers;
— retailer product quality standards deterring smallholders from supplying produce to the market;
— high contractual penalties for partial or total non-delivery of orders by suppliers;
— product take-back clauses in supplier contracts allowing retailers to return product to suppliers once a residual shelf-life has been reached;
— often poor demand forecasting and replenishment systems and a lack of FSC transparency; and
— difficulties inherent in transitioning from trading systems previously driven by spot market prices towards long-term contracts.

Literature on transitional economies lacks analysis of the relative resource efficiency of alternative models of retail development, although there are lessons that might be learnt from industrialized countries. For instance, in the UK (DEFRA 2007), estimated contractual penalties, product take-back clauses and poor demand forecasting had a combined influence that drove 10 per cent over-production and high levels of wastage in the UK FSC.

Accounts of supermarket expansion in some countries suggest there are instances of successful adaptation to traditional supply chains (Chen *et al*. 2005), particularly in regions that have not been so involved in export-orientated markets. Where central wholesale markets are used to source fresh produce, retailers may be reliant on wholesalers to perform the ‘out-grading’ that in developed countries is likely to occur on-farm or at front-end packing operations. In countries with traditional two-tier produce markets (higher quality export and lower quality domestic markets), local supermarkets have created a third market for intermediate to high-quality products. At the same time, retailers provide upward pressure to improve product quality and food safety in the domestic market.

Growth in FFV production has been particularly strong in the Asia-Pacific region (Rolle 2006), although the replacement of traditional markets with supermarkets has been slower in the fresh produce sector, compared with other food sectors. Within the region, FFV producers can be grouped into small farmers, groups of farmers, cooperatives, commercial farmers and foreign entities/multinationals. These producers target different markets, and show a gradient in their production capabilities, access to technologies, markets information and infrastructure. Production is dominated by small farmers with limited access to resources and technology. Growers generally focus on production activities, showing little interest in post-harvest and marketing, which are primarily undertaken by middlemen and traders. Their major markets include highly disorganized traditional wholesale and wet markets, though many supply the requirements of institutions, supermarkets and fast food chains. With limited access to financial resources and low returns from agricultural production, these farmers do not invest in new technologies or improve yields through increasing inputs to production (Mittal 2007).

Development of more industrialized FSCs can also result in growth in the food processing sector. In some BRIC countries, public sector investment is being considered to accelerate this process. In India, the government is discussing an ‘evergreen revolution’, which will involve the build-up of food processing units. While this is a sensitive issue because of concerns about the industrialized sector taking control over small farmers, the improved infrastructure has helped farmers branch out into new foods, diversifying their incomes.

*Industrialized FSCs*: medium-high income countries often argue that better resource efficiency and less waste are achieved through centrally processing food. Although more food wastage occurs at the factory, logic suggests less waste overall is generated as there is less ‘scratch-cooking’ at home. However, research on post-consumer food waste suggests that this is not the case, as consumers still waste significant quantities of food, thus potentially negating the benefits of centralized food processing.

### (c) Estimates of FSC losses

#### (i) Introduction

The distinction between perishable and non-perishable food stuffs is an important consideration in post-harvest losses and the adequacy of FSC infrastructure (table 3). The following sections review post-harvest losses for cereals (non-perishables) and FFVs (perishables); few sources were found for other food types.

**Table 3.** Comparison between non-perishable and perishable food crop properties and storage regimes. From FAO (1981).

non-perishable food crops	perishable food crops
harvest mainly seasonal, need for long-term storage	possibility of permanent or semi-permanent production, short-term storage needs
preliminary treatment (except threshing) of the crop before storage exceptional	processing of dried products—an alternative to the shortage of fresh products
products with low level of moisture content (10–15% or less)	products with high level of moisture in general between 50 and 80%
small ‘fruits’ of less than 1 g	voluminous and heavy fruits from 5 g to 5 kg or more
respiratory activity of stored product very low, heat limited	high or even very high respiratory activity of stored products inducing heat emission in particular in tropical climates
hard tissues, good protection against injuries	soft tissues, highly vulnerable
good natural disposition for storage even for several years	products easily perishable, natural disposition for storage between some weeks and several months
losses during storage mainly from exogenous factors (moisture, insects or rodents)	losses owing partly to endogenous (respiration, transpiration, germination) and to exogenous factors (rot, insects)

#### (ii) Post-harvest loss estimates for non-perishable food crops

Losses in industrialized countries are not included as loss rates are generally considered to be low (e.g. barley losses can be as low as 0.07–2.81%; Smil 2004*a*) and are not considered significant under normal circumstances.

Grain losses occur in post-harvest systems owing to physical losses (spillage, consumed by pests) or loss in quality. Few datasets were found relating to loss of grain quality, owing to difficulty in measurement. As most of the global production of maize, wheat, rice, sorghum and millet must be held in storage for periods from one month to more than a year, many studies focus largely on storage losses.

Data available for rice post-harvest losses, based on field surveys and used here as an example, are quite extensive (table 4) and represent the ‘best case’ compared with data for other crops. More extensive studies suggest that about 15 per cent of grain may be lost in the post-harvest system (Liang *et al*. 1993), with higher storage losses associated with the 80 per cent of China's grain stored by peasants inside their houses or in poorly constructed granaries. The extent to which variations in data presented in table 4 might relate to different levels of post-harvest technology is unclear. For instance, data discussed by Grolleaud's review (2002) found the heaviest losses at the milling stage, perhaps attributable to case studies from more mechanized systems than Liang's data, where storage losses were predominant. This emphasizes the need for post-harvest loss data to be regularly updated and more fully described, particularly for transitioning economies.

**Table 4.** Post-harvest loss estimates for rice.

geographical coverage	estimated % weight loss	comments	source
overall: Asia	13–15	quoted as 15% by Smil (2004*b*)	Grolleaud (2002)
country summaries			
West Africa	6–24	drying 1–2%; on-farm storage 2–10%; parboiling 1–2%; milling 2–10%	FAO (2007)
Malaysia	17–25	central storage 6%; threshing 5–13%	
	approx. 13	drying 2%; on-farm storage 5%; handling 6%	
Philippines	9–34	drying 1–5%; unspecified storage 2–6%; threshing 2–6%	
	up to 30	handling 3–10%	
Thailand	8–14	on-farm storage 1.5–3.5%; central storage 1.5–3.5%	
	12–25	on-farm storage 2–15%; handling 10%	
Brazil	1–30	unspecified storage	
Bolivia	16	on-farm 2%; drying 5%; unspecified storage 7%	
India	3–5.5	improved traditional storage	
	6	unspecified storage	
Philippines	10–37		
Vietnam	10–25	‘typical’ conditions	Phan & Nguyen (1995)
	40–80	‘extreme’ conditions	
China (Zhejiang)	14.81	identified drying and storage phases as two main stages of loss	Grolleaud (2002)
China	5–23	excludes processing losses	Yong *et al*. (1997)

Climatic conditions are also an important consideration in determining the wider applicability of data. In humid climates, rice losses are generally greater at the drying stage (Grolleaud 2002). Hodges (undated) reviewed grain losses in East and South Africa, attempting to compare loss rates in hot humid climates (where open storage structures were required to maintain airflow) and hot dry climates (favouring sealed storage designs). Hodges concluded that data on storage losses were too limited to permit reliable comparisons of loss rates under different climates. In common with other authors, Tyler (1982) suggested that the aggregated data reflecting losses on a worldwide basis are of little value. Long-term studies of post-harvest losses in Zambia and India were identified as using ‘reliable methodology’ and indicative of the fact that when post-harvest losses are determined by field survey, storage and related post-harvest losses are usually lower than previously reported (table 5; Tyler 1982).

**Table 5.** Estimates of post-harvest grain losses in farm-level storage. From Tyler (1982).

estimated weight loss (%)	storage period (months)	cause of loss	grain	country
1.7	7	insects	maize	Zambia
4.3	7	insects, rodents and moulds	rice	India (Andhra Pradesh)
3.5	up to 9	insects and rodents	maize	Kenya
3.2	up to 9	insects	maize	Malawi
1.8	up to 9	insects	maize	Malawi
1.7	up to 9	insects	sorghum	Malawi
5.5	6	insects, rodents and moulds	maize	Nepal

In summary, the main factors contributing to overestimation of grain losses were: (i) where extremes are taken rather than averages: ideally sample size and standard deviation should be quoted with the loss estimate to avoid this; (ii) removal from store over the season are not always accounted for and where they do occur, percentage losses calculated on the basis of grain remaining in store will be overestimates unless an inventory is kept; (iii) treating partial damage as a total loss, when the damaged grain would be used by farmers for home consumption or animal feed; and (iv) potential for double-counting losses at different stages in the post-harvest system.

#### (iii) Post-harvest losses for perishable crops

The causes and rates of post-harvest losses for perishable crops are substantially different from those for grains. Horticultural products generally suffer higher loss rates within industrialized and developing countries, although at different points in the FSC and for different reasons. Table 6 summarizes post-harvest loss estimates for FFVs for both developing and industrialized FSCs.

**Table 6.** Post-harvest loss estimates for fresh fruit and vegetables.

country	commodities	post-harvest losses (%)	reference
Egypt	all fruits	20	Blond (1984)
	all vegetables	30	
	grape	28	
	potato	18	
	tomato	43	
Venezuela	broccoli	49	Guerra *et al*. (1998)
	cauliflower	33	
	celery	48	
	leek	20	
	lettuce	35	
	all FFVs		
India		40	Rolle (2006)
Indonesia		20–50	
Iran		>35	
Korea		20–50	
Philippines		27–42	
Sri Lanka		16–41	
Thailand		17–35	
Vietnam		20–25	
loss estimates: less developed countries (research prior to 1981)			
	carrots	44	National Academy of Sciences report (1978) and cited in FAO (1981)
	potatoes	5–40	
	sweet potatoes	35–95	
	yams	10–60	
	cassava	10–25	
	onions	16–35	
	plantain	35–100	
	cabbage	37	
	cauliflower	49	
	lettuce	62	
	banana	20–80	
	papaya	40–100	
	avocado	43	
	peaches, apricots and nectarines	28	
	citrus	20–95	
	apples	14	
loss estimates: US and UK			
USA	all FFVs	2–23, farm-retail stage	Kader (2005)
UK	all FFVs	approx. 10, farm-retail stage	Garnett (2006)
	‘out-graded’ FFVs	25–40, rejected by supermarkets	Stuart (2009)

Kader (2005) estimated that approximately one-third of all FFVs produced worldwide is lost before it reaches consumers. Losses in the USA are estimated from 2 to 23 per cent, depending on the commodity, with an overall average of 12 per cent. A tentative estimate from the UK suggests losses of 9 per cent (Garnett 2006), but this disregards produce that might be left in the field after failing to meet cosmetic or quality criteria. Although not necessarily a post-harvest loss, out-grading represents a significant aspect of waste that is difficult to quantify and largely anecdotal (Stuart 2009), with some produce likely to enter the food processing sector if it does not meet the criteria. In the EU, quality and size classifications for marketing FFVs have excluded non-conforming produce from the market. Recent moves have relaxed these rules to allow the sale of such FFVs where they are labelled appropriately (EC 2008).

The general difference between developed and developing countries is that FFV infrastructure losses are greater in developing than in developed countries. As with grain, more data on post-harvest losses are required to better understand the current situation, and uncertainties around how post-harvest loss data are extrapolated are broadly similar.

As with grain, there is evidence of overestimation of perishable crop losses from traditional subsistence systems. Within such agriculture, the chain from field-to-consumer is usually short, both in time and distance. Traditional harvesting techniques, e.g. using sticks to harvest papaya and mango, may bruise fruit, with loss implications for more extended supply chains. However, a high proportion of FFVs are consumed because every quality finds a ready consumer within the locality.

Where FFVs are marketed, there is potential for FSCs to be ill-adapted to the changing circumstances. Post-harvest loss literature cites measures to reduce these losses, including gentler handling of produce, better conditioning, faster transportation and proper storage (FAO 1981; Rolle 2006). These measures require improved infrastructure and a heightened interest in quality of produce by the grower. Choudhury (2006) highlights high loss rates associated with a lack of packing houses in India, with FFVs generally packed in the field and some even transported without transit packaging. Furthermore, 30 per cent of FFV production in India is wasted through lack of a cold chain (Mittal 2007). Government-supported cold chain programmes are operational in countries such as Thailand and Taiwan. In lower income countries, low-cost energy-efficient cool storage systems have been developed and implemented in an effort to minimize FFV storage losses.

### (d) Overview of food processing and retail losses: UK example

**Figure 2.**
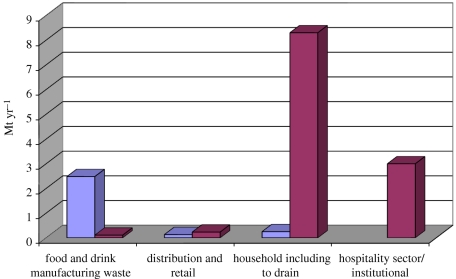
Food waste profile, UK food processing, distribution, retail and post-consumer. Light blue bars, recovery/reuse; magenta bars, disposal. From WRAP (2010).

This review of post-harvest losses has considered wastage rates from the perspective of different food types. For industrialized countries, where waste arising data are compiled, it is possible to quantify total food losses across different sectors of the FSC. Figure 2 provides this profile for the UK, with the post-consumer element included for comparison.

Food and drink waste is estimated to be approximately 14 megatonnes (Mt) in the UK, of which 20 per cent is associated with food processing, distribution and retail. Household food waste makes the largest single contribution, but reliable estimates of other post-consumer wastes (hospitality, institutional sources) have yet to be published. The estimated total waste arisings from the food and drink manufacturing and processing sector is 5 Mt per annum, where approximately 2.6 Mt is estimated to be food waste; a further 2.2 Mt of by-products are diverted into animal feed (WRAP 2010). Waste production surveys have identified that a large proportion of these arisings originate from meat and poultry, FFVs and beverage sectors. These wastes largely consist of by-products and unsold prepared food products.

As an indication of the overall resource efficiency of the sector, a mass balance estimated that nearly 56 Mt of ingredients are used annually to produce 59 Mt of food products (C-Tech 2004). More mass balances conducted at food and drink manufacturing sites suggested that around 16 per cent of raw materials were wasted (WRAP 2010).

A small number of large retailers in the UK exercise market power over the 7000 suppliers within the sector. To avoid being ‘de-listed’, food manufacturers will often over-produce in case extra quantities are required at short notice. For manufacturers of supermarkets' own brands, packaged surplus production cannot be sold elsewhere and becomes waste; however, the sector is adept at reusing the majority of food waste generated (C-Tech 2004). More detailed supply chain mapping studies are under way to understand where the greatest opportunities for increased resource efficiency lie (WRAP 2010).

At the retail and distribution stage, the most recent estimate suggests 366 kt per annum (WRAP 2010). Amounts of waste produced by food retailers vary between outlet types. Small grocery stores produce proportionately more waste than large supermarkets, as the former tend to be used by consumers for top-up shopping, which makes demand unpredictable.

## 3. Post-consumer food waste

This section summarizes knowledge of post-consumer food waste, focusing on household sources and quantities of food wasted. Data from a handful of OECD countries and economies in transition were reviewed. We were unable to find published studies relating to post-consumer food waste in the developing world, where a ‘buy today, eat today’ food culture exists.

### (a) Types of study and data on post-consumer food waste

Methodologies for post-consumer waste analysis vary, from small numbers of households weighing food waste or using kitchen diaries to waste compositional and behavioural studies involving thousands of households (WRAP 2008, 2009*a*). Others have used contemporary archaeological excavations of landfill sites to determine historical levels of food waste (Jones 2006); estimated household food waste indirectly from loss coefficients based upon existing research (Sibrián *et al*. 2006); or estimated wastage using statistical models relating population metabolism and body weight (Hall *et al*. 2009).

Some studies have measured household food waste as a percentage of total consumed calories, others as a percentage of the total weight of consumed food or of the consumed food items. Some studies have sought to estimate the environmental impact of food waste, including the embodied greenhouse gas emissions (WRAP 2008, 2009*a*) or water (Lundqvist *et al*. 2008).

Most of the estimates relying on exogenous food loss coefficients come from studies dating back to the 1970s. Since then, technological progress resulting in fast changes in markets, distribution systems and household storage facilities have rendered these estimates outdated (Kantor 1998; Naska *et al*. 2001). Increased consumer choice and a decrease in the proportion of disposable income spent on food have tended to increase wasteful behaviour. As such, any study where waste was measured over time as a constant proportion of food consumed is in danger of being inaccurate (Sibrián *et al*. 2006).

In many studies, food scraps fed to domestic animals and sink disposals were not included, thus yielding inaccurate estimates for total food waste (Harrison *et al*. 1975; Wenlock & Buss 1977; T. Jones 2003, unpublished data). In some cases, the wastage owing to feeding to pets reached 30 per cent of the total food wastage in dietary energy terms (Mercado-Villavieja 1976; Wenlock *et al*. 1980; Osner 1982).

### (b) Definition of household food waste

**Figure 3.**
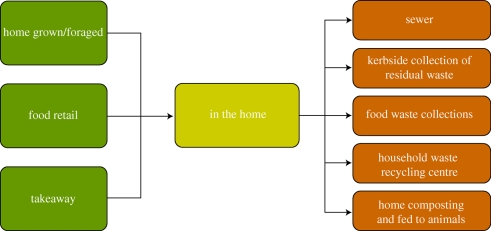
Sources and disposal routes of household food and drink in UK homes. From WRAP (2009*a*) Household food and drink waste in the UK.

**Figure 4.**
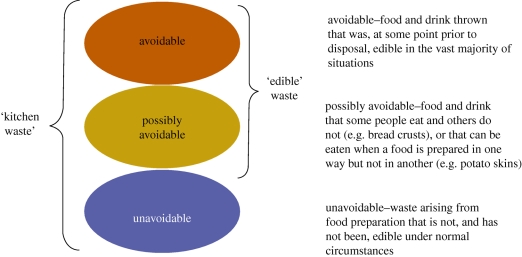
Definitions associated with household food and drink waste. From WRAP (2009*a*) Household food and drink waste in the UK.

Sources of food and drinks that are consumed within the home include retail and contributions from home-grown food and takeaways. Figure 3 indicates which disposal routes are classified as household waste streams. In effect, this excludes significant quantities of food and drink eaten ‘on-the-go’, in the workplace or in catering establishments. Wherever possible, the distinction is made between three classifications of household food waste (figure 4): ‘avoidable’, ‘possibly avoidable’ and ‘unavoidable’.

### (c) How much food is wasted in the home?

#### (i) UK studies

Pre-Second World War studies (Cathcart & Murray 1939) showed that 1–3% of food was wasted in the home in Britain. The next major study, by the UK Ministry of Agriculture, Fisheries and Food in 1976, investigated the 25 per cent ‘crude energy gap’ between estimates of embodied energy in domestically grown and imported food (an average of 12.3 MJ (2940 kcal) of energy to each person per day), and the average physiological requirement for energy according to the UK Department of Health and Social Security (9.6–9.8 MJ (2300–2350 kcal)/person per day) (Wenlock *et al*. 1980; Osner 1982). The resultant survey of 672 households recorded all the potentially edible food wasted in a week, and found that, when assessed against the expected usage of food in the home, wastage accounted on average for 6.5 per cent of the energy intake in summer and 5.4 per cent in winter (Osner 1982).

More recently, the Waste and Resources Action Programme (WRAP) has shown that household food waste has reached unprecedented levels in UK homes (WRAP 2008, 2009*a*,*b*), with 8.3 Mt of food and drink wasted each year (with a retail value of £12.2 billion, 2008 prices) and a carbon impact exceeding 20 Mt of CO_2_ equivalent emissions. The amount of food wasted per year in UK households is 25 per cent of that purchased (by weight).

#### (ii) US studies

A 1998 study by Kantor *et al*. of food waste in the USA revealed that 25 per cent of food was wasted. Archaeological excavations of US landfills by the University of Arizona (Griffin *et al*. 2009) also drew attention to food waste in the USA and provided quantitative data on the likely scale.

Jones *et al*. (T. Jones, A. Bockhorst, B. McKee & A. Ndiaye 2003, unpublished data) estimated that American households discarded 211 kg of food waste per year, not including food to drain, into home composting or feed to pets. The amount of food loss at the household level was estimated to be 14 per cent (T. Jones, A. Bockhorst, B. McKee & A. Ndiaye 2003, unpublished data), costing a family of four at least $589.76 annually (Jones 2004). Jones has estimated that overall food losses in the USA amount to US$90–100 billion a year, of which households threw away US$48.3 billion worth of food each year (Jones 2006). Finally, the US Environmental Protection Agency estimated that food waste in 2008 accounted for 12.7 per cent (31.79 Mt) of municipal solid waste stream (USEPA 2009).

#### (iii) Miscellaneous studies from other countries

Despite the dearth of food waste data in Australia, a submission to the Senate inquiry estimated that food waste comprises 15 per cent of the 20 Mt of waste that goes to landfill each year (Morgan 2009).

A South Korean study (Yoon & Lim 2005) followed their 2002 landfill ban on food waste in the municipal waste stream and suggested that food accounted for 26–27% of household waste (Baek 2009). Despite an awareness-raising effort in advance of the ban, food waste increased by almost 6 per cent over 4 years after the ban, with increased consumption of FFVs linked to higher incomes cited as a reason.

The Dutch Ministry of Agriculture, Nature and Food Quality has estimated that Dutch consumers throw away approximately 8–11% of food purchased (2009), equating to 43–60 kg of food waste with an average value of €270–400 per person per year (Thönissen 2009).

Finally, a UN FAO study (Pekcan *et al*. 2006) estimated household food wastage using a sample of 500 households in Ankara, Turkey, grouped according to socio-economic status. Mean energy intake levels per consumption unit and per person were 2692.6 and 2207.9 kcal d^–1^, respectively. The mean daily energy loss from acquisition of food to plate waste was 481.7 kcal by the average household and 215.7 kcal per person, amounting to 8.9 per cent of daily per person dietary energy consumption. The average daily discards per household and per person were 816.4 and 318.8 g, respectively.

#### (iv) Comparison of food waste arisings between countries

The few quantitative studies that relate to post-consumer food waste are difficult to compare in terms of food wastage per household, as demonstrated in table 7. Different methods and definitions applied to the measurement of food waste reduce comparability of data and some methods do not provide robust estimates owing to small samples. Different definitions of food waste are applied, particularly with regard to ‘edible’ and ‘inedible’ fractions and the extent to which alternative disposal routes are considered. When differences are identified, the post-consumer element must also be considered in the context of the whole FSC.

**Table 7.** Comparison of quantitative estimates for household food waste (kg/household) between five studies.

country	source	methodology	food waste estimate kg per household per year	definition of household food waste				strengths/weaknesses
				non-household municipal waste included?	down drain/home composting/fed to pets included?	edible food waste included?	inedible food waste included?	
USA	Jones (2004)	food sector data combined with compositional analysis, archaeology of landfill sites	212 kg per household per year edible food waste	√	×	√	×	methodology not described in any detail
USA	USEPA (2009)	macro-level mass-flow modelling technique, industrial production data, compositional analysis, 1960–2008	154 kg household food waste (1970) to 233 kg (2004)	√	×	√	√	mass flow depends on assumed relationships between product types and waste, imports and exports to US economy difficult to account for
England	DEFRA (2010)	direct measurement of waste composition integrated with national arisings statistics	240 kg /household/year food waste discarded into municipal waste stream	√	×	√	√	compositional studies do not provide details of wastage of different food types
UK	WRAP (2009*a*)	waste compositional analysis studies and household diaries/surveys	270 kg/household/year edible food and drink waste	×	√	√	×	contains an array of different measurement types, complex to undertake, method supplies detail on causes and types of food waste
Turkey	Pekcan *et al*. (2006)	cross-sectional study of households in Ankara, Turkey, based on questionnaire	298 kg/household/year	×	×	√	√	limited sample and accuracy of recall questionnaire technique

### (d) What types of food are being wasted?

Most studies that have sought to identify the main food types wasted find that it is the most perishable food items that account for the highest proportion of food waste. FFVs are usually among the most-wasted items, followed by other perishables like bakery and dairy products, meat and fish (Pekcan *et al*. 2006; WRAP 2008; Morgan 2009; Thönissen 2009).

There is often a large variation in the wastage rates for different food types: WRAP (2009*a*) found that 7 per cent of milk purchases is wasted, 36 per cent of bakery and over 50 per cent of lettuce/leafy salads (by weight), while Jones *et al*. (T. Jones, A. Bockhorst, B. McKee & A. Ndiaye 2003, unpublished data) found similar variations in the average wastage rates for different food types.

**Figure 5.**
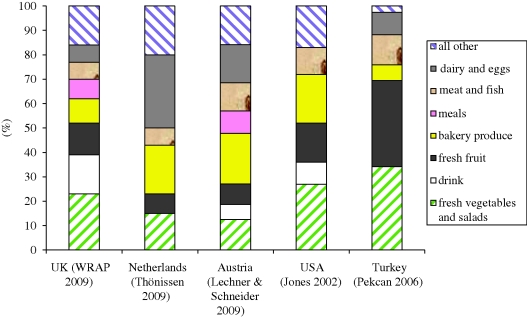
Summary of household food waste composition across five countries.

**Figure 6.**
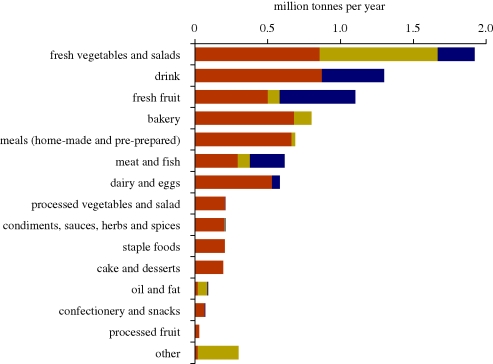
Weight of food and drink waste by food group, split by ‘avoidability’. Brown bars, avoidable; yellow bars, possibly avoidable; dark blue bars, unavoidable. From WRAP (2009*a*) Household food and drink Waste in the UK. Brown bars, avoidable; yellow bars, possibly avoidable; dark blue bars, unavoidable.

Although food and drink categories are not fully consistent across studies, figure 5 serves to highlight variation in household food waste composition. Thönissen (2009) found an unusually high proportion of food waste consisted of dairy products, while in the Turkish data, wasted FFVs accounted for the highest proportion (Pekcan *et al*. 2006). The extent to which such differences relate to consumption patterns or different wastage rates cannot be divined from these data alone, although the Turkish study noted the importance of fruit in the diets of households studied. Nor do these compositional data distinguish between avoidable and unavoidable food waste, the exception being the UK data shown in figure 6.

### (e) Who is wasting all of this food?

The following factors may help to explain variation in quantities of household food waste generated.

— *Household size and composition*. Studies from the UK (Wenlock & Buss 1977; Osner 1982; WRAP 2009*a*) and the USA (Van Garde & Woodburn 1987) show that food wastage was significantly influenced by the composition of the family, with adults wasting more in absolute terms than children, and larger households wasting less per person than smaller households. Single-person householders tend to throw away more *per capita*, and households with children tend to waste more than households without children, although rates vary with the children's age.
— *Household income*. The majority of studies suggest that there is lower food loss in low-income than in high-income households (Osner 1982; Brook Lyndhurst 2007); some studies (Dowler 1977; Wenlock *et al*. 1980; T. Jones 2003, unpublished data) found little or no correlation between income and food wastage.
— *Household demographics*. Studies in the UK (Osner 1982; Brook Lyndhurst 2007) and Australia (Hamilton *et al*. 2005) suggest that young people waste more than older people, with pensioner households wasting the least (such households normally contain comparatively fewer people).
— *Household culture*. There is some indication that culture partly determines food wastage. For example, Hispanic households in the USA have lower food loss rates (approx. 25% less) than non-Hispanics and Hispanic households consume more FFVs compared with non-Hispanic households. However, FFV consumption among Hispanic households has decreased over the last 20 years as they consume more prepared foods.

Food waste studies in the UK (Brook Lyndhurst 2007) and Australia (Hamilton *et al*. 2005) have sought to profile segments of the population according to their attitudes and behaviours with respect to food, drawn from household survey work and waste compositional data. Such studies are a useful tool in targeting particular elements of the population with waste reduction messages and information.

### (f) Why are we wasting all of this food?

There are a limited number of studies focusing specifically on the reasons for householders wasting food, largely restricted to the UK (Exodus 2006; Brook Lyndhurst 2007; WRAP 2008, 2009*a*,*b*), the USA (Van Garde & Woodburn 1987) and Australia (Hamilton *et al*. 2005).

**Figure 7.**
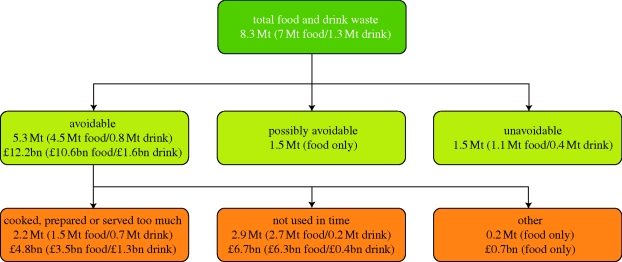
Classification of UK household food and drink waste by avoidability, reason for disposal and economic value. Bracketed figures show the tonnages and economic values for food and drink separately. From WRAP (2009*a*) Household food and drink waste in the UK.

These studies highlight a complex array of consumer attitudes, values and behaviours towards food and how varying degrees of food knowledge affect individual's propensity to waste food. It is possible to group identified attitudes, values and behaviours by using a combination of qualitative and quantitative consumer research techniques thereby determining key claimed behaviours, and waste compositional analysis. The resultant information is then used to establish those attitudes, values and behaviours that are the strongest drivers of household food waste. In the UK, detailed research findings described in figure 7 for the two principal reasons why avoidable food waste occurs are: ‘food is not used in time’ and ‘too much food is cooked, prepared or served’.

Bracketed figures show the tonnages and economic values for food and drink separately.

These two broad categories are explained below:

— *Cooked, prepared or served too much*. In the majority of cases, this is because too much food was ‘processed’ in the home, but also covers cases where food was damaged during processing (e.g. burning food). This category could be referred to as ‘leftovers’.
— *Not used in time*. This covers food and drink wasted because it passed a date label (e.g. a ‘use by’ or ‘best before’ date), has gone mouldy or looked, smelt or tasted bad.

From the research conducted in the UK (table 8), Australia and the USA, some conclusions can be drawn about the main factors that drive food waste in the home and practical solutions can be shaped that make it easy for consumers to reduce the amount of food they waste.

**Table 8.** Summary of UK consumer research on main contributory factors to food being wasted. Note: % have been rounded.

stages in the household food ‘journey’	main contributory factors/behaviours leading to food waste	% respondents admitting this behaviour
poor pre-shop planning	failure to check stocks in cupboards, fridges and freezers prior to shopping	14^a^
	failure to prepare an adequate shopping list	sometimes: 40^a^; never: 19^a^
in-store behaviour	not sticking to a shopping list	always or sometimes: 52^a^; never: 8^a^
	impulse buying (buying items they had not intended to)	74^a^ (half of unplanned purchases as a result of retail promotions)
food date labels	not understanding meaning of ‘use by’ date	45^b^
	not understanding meaning of ‘best before’ date	49^b^
	confusing ‘best before’ date with ‘use by’ date (with potential for food to be thrown away unnecessarily)	36^b^
	high sensitivity to food hygiene (will not take chance with food close to ‘best before’ date)	20^c^
storing food correctly	food ‘gone off’ or mouldy	33^a^
	not knowing to maintain fridge temperature at 5°C	60^a^
meal planning	failure to plan meals	all of the time: 66^a^; some of the time: 42^a^
food management in home	food not used before going past ‘use by’ or ‘best before’ date	60^a^
portion control	preparing meal portions that are too large	40^a^
poor ‘home economics’ skills	from pre-shop planning to recombining leftovers into new meals	75^c^

^a^Exodus (2006; *n* = 2939 UK households).

^b^FSA (2008; *n* = 2627 UK adults).

^c^Brook Lyndhurst (2007; *n* = 1862 GB residents aged 16+).

## 4. Food waste to 2050: projections and uncertainties

Food wasted along the FSC is the outcome of many drivers: the market economy, resource limitations and climate, legislation and cultural differences being just a few. We have outlined the difficulties in defining and quantifying such waste and described how production of waste differs within the developing, transitional and developed worlds. Here we discuss the trends likely to drive waste production in future, where there could be the greatest potential for reduction of food waste to occur in developing and developed worlds, and what policies and systems may be required to reduce food waste to 2050.

### (a) Future trends

In the developing world, lack of infrastructure and associated technical and managerial skills in food production and post-harvest processing have been identified as key drivers in the creation of food waste, both now and over the near future (WFP 2009). This situation contrasts with that in developed countries where our interviewees forecast the majority of food waste continuing to be produced post-consumer, driven by the low price of food relative to disposable income, consumers' high expectations of food cosmetic standards and the increasing disconnection between consumers and how food is produced. Similarly, the increasing urbanization within transitioning countries will potentially disconnect those populations from how food is grown, which is likely to further increase food waste generation.

Across the globe, resource and commodity limitations, in part as a result of an increasing population but also owing to impacts of climate change, were viewed as being likely to increase the economic value of food, potentially driving more efficient processes that could lead to food waste reduction. Industrialized FSCs will continue to develop in response to these wider challenges by the development of shared logistics (e.g. collaborative warehousing), identification and labelling of products (use of barcodes and RFID tags) and better demand forecasting (Global Commerce Initiative 2008), and domestic kitchen technologies (smart fridges, cookers, online meal planning and recipe resources) may make it easier for consumers to manage their food better and waste less of it.

### (b) The greatest potential for food waste reduction

Interviewees emphasized the importance of implementing sustainable solutions across the *entire* FSC to fully realize the potential for food waste reduction. In developing and emerging economies, this would require market-led large-scale investment in agricultural infrastructure, technological skills and knowledge, storage, transport and distribution. Such investments have been shown to stimulate rural economies (WFP 2009), e.g. the development of the Nile Perch Fishery in East Africa. In this case, and despite the unintended consequences of over-fishing and disruption of local communities, the international market for Nile perch stimulated infrastructure development and considerably reduced post-harvest losses.

Where international markets and local policies and investment are lacking, large-scale capital investment in infrastructure in developing countries has often failed (FAO 2003; Kader 2005). For long-term sustainability, development across the FSC in the developing world requires locally supported government policies and investment alongside any market-led private investment with reach through into developed world markets. Examples of integrated cross-FSC approaches to food waste reduction include various cooperative schemes, e.g. the Common Code for Coffee Community, and the Sustainable Agriculture Initiative.

Conversely, the greatest potential for the reduction of food waste in the developed world lies with retailers, food services and consumers. Cultural shifts in the ways consumers value food, stimulated via education, increased awareness of the FSC and food waste's impact on the environment have the potential to reduce waste production. Improved food labelling and better consumer understanding of labelling and food storage also have food waste reduction potential. WRAP's ongoing activities in this area, through programmes such as ‘Love Food Hate Waste’, are very recent and their impact is yet to be established. With food price recognized as the most important factor in determining consumer decisions, anecdotal evidence suggests that the economic crisis has stimulated a shift in consumer attitude to food waste.

Innovative technology throughout the FSC, in both developed and developing worlds, particularly in packaging, contributes to improving shelf life for perishable foods and semi-prepared meals. Continued developments in packaging, e.g. utilizing nanotechnology and materials science, have the potential to further increase shelf life.

### (c) Policy, systems and practices

In the developing world, transfer of existing technologies and the spread of good practice, allied to market-led investment, have the greatest potential to reduce food waste across the FSC. It is of key importance, however, that practical developments address the problems of local farmers, using indigenous knowledge where that has been shown to be sustainable. Without participation of local farmers, such knowledge transfer is unlikely to succeed.

While attempts to shift consumer behaviour *may* result in reduction in food waste in developed countries, changes in legislation and business behaviour towards more sustainable food production and consumption will be necessary to reduce waste from its current high levels. An example might be through the development of closed-loop supply chain models (WEF 2010). In such models, waste of all forms would be fed back into the value chain (such as packaging waste being re-used), food graded as lower quality for cosmetic reasons and food that is surplus to retailer or manufacturers, to be made available through alternative routes (e.g. Fareshare or as cheaper alternatives), while unavoidable food waste would be utilized as a by-product, e.g. in providing energy from waste using the appropriate technology.

## 5. Conclusions

A firm evidence base from which to assess food waste globally is lacking, with no specific information on the impact of food waste in BRIC countries a major concern, and with much of the loss estimates from developing countries collected over 30 years ago. There is a pressing need for quantitative evidence covering developing countries and the rapidly evolving BRIC country FSCs. Without systematic evidence, the arguments over the potential for reducing global food waste as a contribution to feeding nine billion people by 2050 will remain largely rhetorical, and measuring progress against any global reduction target impossible.

As a consequence of the information gaps and uncertainties, there is no consensus on the proportion of global food production that is currently lost. Ranges between 10 and 40 percent of total global food production and as high as 50 per cent are quoted, but on closer examination, these estimates all link back to the same limited primary datasets, where much of the published data relates to fieldwork undertaken in the 1970s and 1980s. Recent reviewers of these data claim there is a tendency to over-state losses in relation to traditional agricultural systems in developing countries, a point reiterated in this review.

The lack of infrastructure in many developing countries and poor harvesting/growing techniques are likely to remain major elements in the generation of food waste. Less than 5 per cent of the funding for agricultural research is allocated to post-harvest systems (Kader 2003), and yet reduction of these losses is recognized as an important component of improved food security (Nellemann *et al*. 2009). Irrespective of global region, there is a need for successful introduction of culture-specific innovations and technologies across the FSC to reduce losses.

Linked to the above, market transformation has enormous potential to develop FSC infrastructure and reduce waste in developing and BRIC countries. Account should be taken of the impact of market transformation on the local communities to whom food may no longer be available.

The rapid expansion of FFVs supplied to consumers in transitional countries is highly likely to have resulted in significant post-harvest losses, owing to inadequate infrastructure. In the industrialized world meanwhile, post-harvest losses have been squeezed out of grain supply through heavy technological investments, while for FFVs, retailers' and consumers' demand for ‘cosmetically perfect’ produce has created significant post-harvest losses through ‘out-grades’. There is also strong evidence of an increase in post-consumer waste over the past several decades, particularly in the developed world, with pockets of data supporting similar behaviour in BRIC countries.

The majority of studies show that as the proportion of income spent on food declines, food waste increases. There is clear evidence of a distribution of waste across demographic groups, with the lowest wastage rates in the immediate post-war age generation. However, it would be a mistake to assume that the demographic distribution will remain the same in the future, as today's elderly generally exhibit a ‘waste not want not’ mentality, while the elderly of the future are likely to continue to retain the same attitudes and behaviours to food that they have today.

There are clearly fundamental factors affecting post-consumer food waste worldwide, some of which may require solutions that involve direct communication and awareness-raising among consumers of the importance of reducing food waste. Others require government interventions and the support and cooperation of the food industry itself, such as improving the clarity of food date labelling and advice on food storage, or ensuring that an appropriate range of pack or portion sizes is available that meets the needs of different households.
